# Denture Wearing Moderates the Association between Aspiration Risk and Incident Pneumonia in Older Nursing Home Residents: A Prospective Cohort Study

**DOI:** 10.3390/ijerph16040554

**Published:** 2019-02-14

**Authors:** Kenji Takeuchi, Maya Izumi, Michiko Furuta, Toru Takeshita, Yukie Shibata, Shinya Kageyama, Yuka Okabe, Sumio Akifusa, Seijun Ganaha, Yoshihisa Yamashita

**Affiliations:** 1 Section of Preventive and Public Health Dentistry, Division of Oral Health, Growth and Development, Faculty of Dental Science, Kyushu University, Fukuoka 812-8582, Japan; k-takeuchi64@umin.ac.jp (K.T.); r15izumi@fa.kyu-dent.ac.jp (M.I.); mfuruta@dent.kyushu-u.ac.jp (M.F.); taketooo@dent.kyushu-u.ac.jp (T.T.); yukie@dent.kyushu-u.ac.jp (Y.S.); s.kageyama@dent.kyushu-u.ac.jp (S.K.); okabe0830@dent.kyushu-u.ac.jp (Y.O.); r11akifusa@fa.kyu-dent.ac.jp (S.A.); 2 OBT Research Center, Faculty of Dental Science, Kyushu University, Fukuoka 812-8582, Japan; 3 Department of Oral Functional Management, School of Oral Health Sciences, Faculty of Dentistry, Kyushu Dental University, Fukuoka 803-8580, Japan; 4 Kizuna-kai, Aso-kizuna Dental Clinic, Kumamoto 869-2612, Japan; asokizunashika@lapis.plala.or.jp

**Keywords:** dental prosthesis, dysphagia, elderly, oral health, swallowing

## Abstract

Aspiration is increasingly recognized as a major risk for pneumonia, but a potential link between wearing dentures and incident pneumonia with aspiration risk is unclear. The aim of this study was to investigate whether denture wearing moderates the association between aspiration risk and incident pneumonia in older adults. We used prospective cohort data of 156 residents aged >70 years from eight nursing homes in Aso, Japan. Aspiration risk was evaluated using the modified water swallowing test. During a 1-year follow-up (2014 to 2015), information on incident pneumonia was obtained from nursing home medical records. During follow-up, pneumonia developed in 7.1% of participants. In the multivariate-adjusted Cox proportional hazards model, after adjusting for potential confounders, aspiration risk was independently associated with a 4.4-fold higher hazard ratio (HR) of incident pneumonia (95% confidence interval, CI, 1.16–16.43). The difference in the risk of incident pneumonia between subjects with aspiration risk who were wearing dentures and those not at risk of aspiration was not significant, whereas those with aspiration risk without dentures had a 7.3-fold higher HR of incident pneumonia than those not at risk of aspiration (95% CI, 1.02–52.63). Denture wearing might partially moderate the increased risk of incident pneumonia associated with aspiration risk.

## 1. Introduction

Globally, pneumonia, together with other lower respiratory tract infections, was rated the fourth leading cause of death in 2010, with an estimated 2.81 million deaths [1]. In Japan, a fast-aging society, while pneumonia had previously occupied the fourth position since 1975, it supplanted hypertension as the third leading cause of death in 2011 [2]. The risk of death from pneumonia increases with aging [3]. Furthermore, the incidence rate of pneumonia is known to be highest in nursing home residents [4]. Therefore, it is important to identify modifiable risk factors for the incidence of pneumonia in older nursing home residents. 

Aspiration of food, reflux, and oral bacteria into the lower respiratory tract is recognized as the most common pathogenic mechanism for pneumonia among older adults [5]. In addition, aspiration can be induced as a consequence of decline in swallowing function with aging [6]. This suggests that aspiration risk, reflecting poor swallowing function, may accelerate the incidence of pneumonia. On the other hand, it has been suggested that wearing dentures may have a preventative impact on aspiration risk [7]. We reported that even if individuals have multiple tooth loss, those with restoration of posterior teeth occlusion by wearing dentures had a lower aspiration risk than those with loss of posterior teeth occlusion [7]. Furthermore, wearing dentures not only contributes to oral health for the maintenance and restoration of chewing function, but also may have implications for maintaining general health, such as cognitive and physical function [8,9]. These findings imply a possible link between wearing dentures and incident pneumonia with aspiration risk. Therefore, the purpose of this study was to investigate whether denture wearing moderates the association between aspiration risk and incident pneumonia in older nursing home residents using a prospective cohort study design.

## 2. Materials and Methods 

### 2.1. Study Design and Population

This 1-year prospective cohort study was carried out in eight nursing homes in Aso City, Kumamoto Prefecture, Japan. The baseline survey was conducted in February to June 2014, and the follow-up survey was conducted 1 year later. Older nursing home residents were enrolled in this study along with their surrogate decision-makers. Residents on feeding tubes and those being cared for at a hospital were excluded. At baseline, 279 residents participated in a swallowing function assessment and an oral examination and completed a comprehensive questionnaire survey for the study. After excluding 60 subjects with more than 10 teeth at the baseline examination, 5 subjects who were previously diagnosed with Parkinson’s disease, and 58 subjects with missing responses to survey questions on other covariates used in the analysis, the remaining 156 subjects (35 men, 121 women) were enrolled in the final cohort. The study protocol was approved by the Kyushu University Institutional Review Board for Clinical Research (25-335). The study population was then followed up until the date of death, the date of leaving nursing homes, or the end of a 1-year follow-up, whichever occurred first. During follow-up, 39 subjects died and 30 subjects left nursing homes. No subjects were lost to follow-up. The study protocol was approved by the Kyushu University Institutional Review Board for Clinical Research. After explaining the purpose and procedure of the study, written informed consent was obtained from all subjects or their surrogates or legal representatives.

### 2.2. Oral Health Status Assessment

At baseline, clinical oral health examinations were carried out by one trained dentist. The examinations included a review of the number of teeth present, denture wearing, and oral hygiene for each subject. The present teeth were defined as healthy, carious, or treated (including crowned, inlay, and abutment teeth for prostheses), inclusive of completely erupted third molars. Unerupted or congenitally missing teeth, root tips, and extremely mobile teeth that were indicated for extraction were not included as remaining teeth. We excluded from the primary analyses those subjects with 10 or more teeth owing to difficulties in properly judging whether wearing dentures was needed from the viewpoint of improvement in chewing function. The plaque index score was used as a measurement of the state of oral hygiene [10]. 

### 2.3. Aspiration Risk Screening

At baseline, aspiration risk screening was clinically performed by a trained dental hygienist using the Modified Water Swallowing Test, which had a sensitivity of 70% and a specificity of 88% for predicting aspiration [11]. Three milliliters of cold water were injected onto the floor of the subject’s mouth with a 5-mL syringe, after which the subject was instructed to swallow, and his/her swallowing was scored as follows: score 1, inability to swallow with choking and/or breathing changes; score 2, swallowing occurred, but with breathing changes; score 3, swallowing occurred with no breathing changes, but with choking and/or wet hoarseness; score 4, successful swallowing with no choking or wet hoarseness; and score 5, in addition to meeting the requirements of score 4, deglutition (dry swallowing) occurred more than twice within 30 s. If the score was ≥4, the test was repeated twice, and the lowest score was used as the test score. A score of ≤3 indicates a risk of aspiration.

### 2.4. Measurement of Other Risk Factors

A wide range of covariates was included in the analyses as potential confounding risk factors, based on previous literature. At baseline, information on demographic characteristics, socioeconomic status, and health conditions was obtained from subjects, or legally acceptable representatives and caregivers, by nursing home care staff using a standardized interview questionnaire, and from information in nursing home medical records. Sex and age were used as demographic characteristics. Occupation was used to stratify subjects according to socioeconomic status, as follows: blue-collar workers, and others. Body mass index (BMI), basic activities of daily living (ADLs), and comorbid conditions were used as health conditions. Body height and weight were measured, and the BMI (kg/m^2^) was calculated and dichotomized as <18.5 (underweight) or ≥18.5 (non-underweight), according to the World Health Organization classification. The Barthel Index was used to measure basic ADLs, assessing independence in self-care activities of daily living, such as transferring, walking up- and downstairs, toilet use, dressing, feeding, and bathing [12]. These scores ranged from 0 to 100, with higher scores representing greater independence. Based on the Barthel Index, we divided the subjects into two categories: >60 (higher independence for basic ADL) and ≤60 (lower independence for basic ADL). The Charlson Comorbidity Index (CCI) was used to measure comorbid conditions, which comprises a sum of 19 physician-diagnosed diseases, such as dementia or diabetes mellitus, with assigned values [13]. 

### 2.5. Outcome Measurement

The primary outcome of the study was the incidence of pneumonia, which was obtained from information in nursing home medical records. During the study period, all subjects were followed up for incident pneumonia.

### 2.6. Statistical Analyses

Baseline subject characteristics according to aspiration risk and denture wearing were evaluated using the Pearson’s chi-squared test for categorical variables and the Mann–Whitney *U* test for continuous variables. Subjects were censored at date of death, leaving from nursing homes, or end of follow-up for survival analyses. Incidence rates of pneumonia were calculated using the person-year method and the cumulative incidences of pneumonia according to aspiration risk and denture wearing were assessed using Kaplan–Meier survival function estimates and log-rank tests.

To evaluate the relationships of aspiration risk and denture wearing with incident pneumonia, we estimated crude and adjusted hazard ratios (HRs) and their 95% confidence intervals (CIs) using a Cox proportional hazards model. In multivariable adjustment, all covariates were included in the model. Additionally, we evaluated whether a higher risk of incident pneumonia among subjects with aspiration risk would persist regardless of whether they wear dentures. For this, the study subjects were divided into the following three categories: (1) ‘non-aspiration risk’, (2) ‘aspiration risk with denture wearing’, and (3) ‘aspiration risk without denture wearing’. The above-mentioned Cox proportional hazards models were used to calculate the HRs and 95% CIs for incident pneumonia to compare the two categories of aspiration risk with the non-aspiration risk category.

All analyses were performed using SPSS statistical software version 24 (IBM Corp., Armonk, NY, USA). Two-sided *p*-values <0.05 were considered statistically significant in all cases. We followed the Strengthening the Reporting of Observational Studies in Epidemiology statement guidelines for the analysis of observational data [14]. 

## 3. Results

Baseline characteristics of the study subjects (*n* = 156; mean age, 89.4 (standard deviation, 6.6) years) according to aspiration risk and denture wearing are shown in Table 1. Subjects at risk of aspiration had significantly higher CCI scores and a lower prevalence of denture wearing than those not at risk of aspiration. Denture wearing was also significantly associated with a lower prevalence of having a BMI <18.5. 

**Figure 1:**
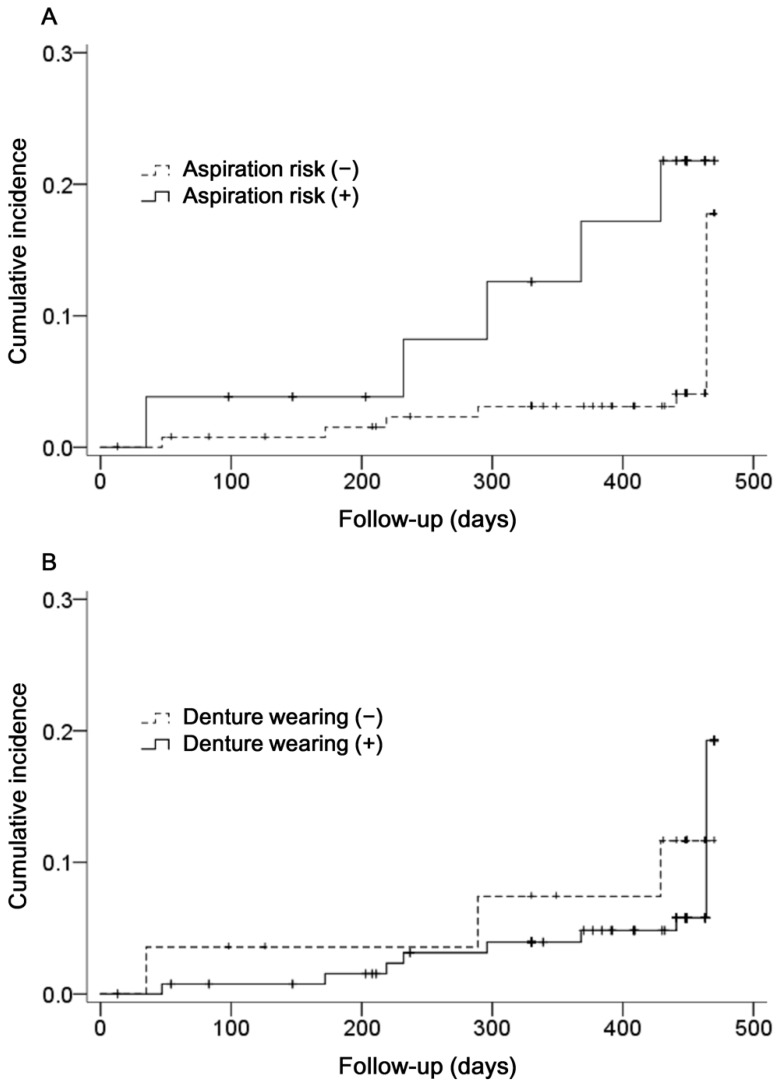
Kaplan–Meier curves showing cumulative incidence of pneumonia according to (**A**) aspiration risk and (**B**) denture wearing.

The data covered 173 person-years (median 448 days per participant; interquartile range 414–449 days). During the follow-up, pneumonia developed in 11 subjects (7.1% of participants). Kaplan–Meier curves showing the cumulative incidence of pneumonia are presented in Figure 1. There was a significant difference in the rate of pneumonia between subjects at risk of aspiration and those not at risk of aspiration. (log-rank test, *p* = 0.002 Figure 1A). Although the rate of pneumonia was greater in denture wearing subjects than those who did not wear dentures, a significant difference was not observed (log-rank test, *p* = 0.339; Figure 1B). 

The crude incidence rates and estimated HRs and 95% CIs of incident pneumonia according to aspiration risk and denture wearing are shown in Table 2. Aspiration risk was significantly associated with greater risk of development of pneumonia (HR, 5.20; 95% CI, 1.58–17.04). This association remained significant after adjustment for all covariates (HR, 4.36; 95% CI, 1.16–16.43). Subjects who did not wear dentures were more likely to develop pneumonia than those wearing dentures, but this tendency did not reach statistical significance in both crude and multivariable-adjusted analyses. 

Additionally, we examined whether denture wearing affected incident pneumonia in subjects with aspiration risk, comparing the risk of development of pneumonia for each of the ‘aspiration risk with denture wearing’ and ‘aspiration risk without denture wearing’ subgroups with the risk of development of pneumonia for the ‘non-aspiration risk’ group (Table 3). In the multivariate-adjusted Cox proportional hazards model, subjects with aspiration risk without denture wearing had a significantly greater risk of the incidence of pneumonia than those not at risk of aspiration (HR, 7.34; 95% CI, 1.02–52.63), but there was no significant difference in risk of the incidence of pneumonia between those with aspiration risk who were wearing dentures and those not at risk of aspiration.

## 4. Discussion

In this prospective cohort study of Japanese nursing home residents, we found that aspiration risk was significantly associated with an increased risk of incident pneumonia, in agreement with the findings of previous studies [15,16,17,18,19]. However, even among subjects with aspiration risk, the risk for those who were wearing dentures was not significantly higher. Therefore, it is reasonable to suppose that wearing dentures may partially moderate the expected increase in incident pneumonia associated with aspiration risk. In this study, almost half of the subjects with aspiration risk did not wear dentures; hence, dental providers and nursing home staff should be alert to the residents having few teeth without denture, while intensified public health efforts should be made to disseminate information about the importance of not only preventing aspiration but also wearing denture for reduction of future incidence of pneumonia. 

There are several possible pathways to explain the link between denture wearing and the development of pneumonia. First, if individuals with few teeth (e.g., <10 teeth) were not wearing dentures, their oral self-cleaning ability might decrease because a decline in salivary secretion is associated with reduced masticatory performance in older adults [20,21]. This reduction in self-cleaning performance can result in increments of potential respiratory pathogens (e.g., food debris and biofilm) on the surface of oral and pharyngeal mucosa. Thus, together with dysphagia, if the aspirated respiratory pathogens into the lungs are large enough, aspiration pneumonia may follow. Second, a recent report has suggested that absence of dentures affects oral and pharyngeal anatomy for swallowing because of a loss of occlusal contact, causing a reduction in swallowing reserve [22]. This alteration in the swallowing mechanism makes older adults more susceptible to dysphagia, which can in turn contribute to developing pneumonia. Our previous report supports this pathway, reporting that having few teeth is correlated with dysphagia, but that wearing dentures can improve swallowing function [23]. 

With regard to the negative aspect of denture wearing, denture wearing during sleep was reported to be associated with an increased risk of incident pneumonia [24]. Similarly, in a recent paper, the application of a quantitative molecular approach showed that about two-thirds of denture wearers harbored significant quantities of known respiratory pathogens, thus increasing the theoretical risk of incidence of aspiration pneumonia [25]. On the other hand, our findings demonstrated that denture wearers had lower incident rates of pneumonia than non-wearers. In this study setting, according to the guideline based on evidence supporting a link between continuous denture wearing and poor denture hygiene, oral candidiasis, and denture stomatitis [26,27], nursing home care staff recommended the removal of dentures during sleep and denture cleaning at least once a day to denture users. Furthermore, we confirmed that all subjects (complete denture subjects, 55.1%) removed their dentures during the night and almost 100% (99.2%) of subjects cleaned their dentures at least once a day themselves or had them cleaned by another person (data not shown).

The main strengths of the present study are the prospective cohort design and the complete follow-up of the subjects. In addition, this is the first reported study to demonstrate a preventive role of denture wearing against incidence of pneumonia resulting from aspiration risk, adjusting for potential confounding factors. On the other hand, some potential limitations of the present study should be noted. A weakness of our study is that the data used in our analysis were based on a relatively small sample taken from older nursing home residents in one municipality in Japan. Accordingly, our findings should be carefully interpreted when generalizing them across all nursing home settings or all older adults. In addition, although many potential confounders were controlled for, the observational study design did not allow for complete exclusion of residual confounding. Furthermore, the lack of information about denture materials (e.g., disinfecting acrylic resin) and classification of dentures based on the distribution of edentulous spaces may have reduced the accuracy of our findings to some extent. 

## 5. Conclusions

The present study demonstrated that aspiration risk is an independent risk factor for incident pneumonia in older nursing home residents in Japan and that denture wearing may be a way to moderate the increased risk of incident pneumonia resulting from aspiration risk. Our findings highlight denture wearing as a possible preventive factor against the burden of pneumonia in aged populations. Further studies are required to understand the mechanisms by which denture wearing affects the risk for aspiration and subsequent pneumonia.

## Figures and Tables

**Table 1:** Baseline characteristics according to aspiration risk and denture wearing.

Characteristic	Aspiration Risk			Denture Wearing		
	Non-Risk, *n* = 132	At Risk, *n* = 24	*p*-Value	Yes, *n* = 128	No, *n* = 28	*p*-Value
Women, %	80.3	62.5	0.065	75.8	85.7	0.323
Age, years	90.5 (71.0–103.0)	90.0 (73.0–100.0)	0.292	90.0 (71.0–103.0)	90.5 (80.0–100.0)	0.873
Blue-collar workers, %	56.1	62.5	0.656	57.0	57.1	1.000
Body mass index <18.5, %	19.7	33.3	0.177	14.8	53.6	<0.001
Barthel Index ≥60, %	16.7	4.2	0.206	16.4	7.1	0.375
Charlson Comorbidity Index	2.0 (0.0–6.0)	2.5 (1.0–6.0)	0.034	2.0 (0.0–6.0)	2.0 (1.0–6.0)	0.360
Plaque index score ≥1, %	31.8	41.7	0.355	32.0	39.3	0.509
Aspiration risk, %	–	–	–	10.2	39.3	<0.001
Denture wearing, %	87.1	54.2	<0.001	–	–	–

Categorical variables expressed as percentage; continuous variable, as median (range).

**Table 2:** Development of pneumonia according to aspiration risk and denture wearing.

	Aspiration Risk		Denture Wearing	
	Non-Risk, *n* = 132	At Risk, *n* = 24	Yes, *n* = 128	No, *n* = 28
Person-years at risk	148.2	24.4	143.4	29.3
Crude incidence rate ^a^	4.0	20.5	5.6	10.2
Crude HR (95% CI)	1.00 (reference)	5.20 (1.58–17.04)	1.00 (reference)	1.89 (0.50–7.14)
Adjusted HR (95% CI) ^b^	1.00 (reference)	4.36 (1.16–16.43)	1.00 (reference)	2.67 (0.54–13.24)

HR = hazard ratio; CI = confidence interval. ^a^ Per 100 person-years. ^b^ Adjusted for sex, age, occupation, body mass index, Barthel Index, Charlson Comorbidity Index, plaque index score.

**Table 3:** Relationship between denture wearing and incident pneumonia stratified according to aspiration risk.

	Non-aspiration Risk, *n* = 132	Aspiration Risk with Denture Wearing, *n* = 13	Aspiration Risk without Denture Wearing, *n* = 11
Person-years at risk	148.2	13.1	11.3
Crude incidence rate ^a^	4.0	22.9	17.6
Crude HR (95% CI)	1.00 (reference)	5.53 (1.37–22.24)	4.77 (0.95–23.91)
Adjusted HR (95% CI) ^b^	1.00 (reference)	3.47 (0.75–16.03)	7.34 (1.02–52.63)

HR = hazard ratio; CI = confidence interval. ^a^ Per 100 person-years. ^b^ Adjusted for sex, age, body mass index, occupation, Barthel index, Charlson comorbidity index, plaque index score.

## References

[1] Lozano R., Naghavi M., Foreman K., Lim S., Shibuya K., Aboyans V., Abraham J., Adair T., Aggarwal R., Ahn S.Y. (2012). Global and regional mortality from 235 causes of death for 20 age groups in 1990 and 2010: A systematic analysis for the global burden of disease study 2010. Lancet.

[2] Cabinet Office (2015). Government of Japan: Aging Society White Paper.

[3] Welte T., Torres A., Nathwani D. (2012). Clinical and economic burden of community-acquired pneumonia among adults in Europe. Thorax.

[4] Loeb M., McGeer A., McArthur M., Walter S., Simor A.E. (1999). Risk factors for pneumonia and other lower respiratory tract infections in elderly residents of long-term care facilities. Arch. Intern. Med..

[5] Janssens J.P., Krause K.H. (2004). Pneumonia in the very old. Lancet Infect. Dis..

[6] Marik P.E., Kaplan D. (2003). Aspiration pneumonia and dysphagia in the elderly. Chest. J..

[7] Okabe Y., Takeuchi K., Izumi M., Furuta M., Takeshita T., Shibata Y., Kageyama S., Ganaha S., Yamashita Y. (2017). Posterior teeth occlusion and dysphagia risk in older nursing home residents: A cross-sectional observational study. J. Oral Rehabil..

[8] Takeuchi K., Izumi M., Furuta M., Takeshita T., Shibata Y., Kageyama S., Ganaha S., Yamashita Y. (2015). Posterior teeth occlusion associated with cognitive function in nursing home older residents: A cross-sectional observational study. PLoS ONE.

[9] Takeuchi K., Izumi M., Furuta M., Takeshita T., Shibata Y., Kageyama S., Ganaha S., Yamashita Y. (2017). Association between posterior teeth occlusion and functional dependence among older adults in nursing homes in Japan. Geriatr. Gerontol. Int..

[10] Löe H. (1967). The gingival index, the plaque index and the retention index systems. J. Periodontol..

[11] Tohara H., Saitoh E., Mays K., Kuhlemeier K., Palmer J.B. (2003). Three tests for predicting aspiration without videofluorography. Dysphagia..

[12] Mahoney F.I., Barthel D.W. (1965). Functional evaluation: The Barthel Index. A simple index of independence useful in scoring improvement in the rehabilitation of the chronically ill. Md State Med. J..

[13] Charlson M.E., Pompei P., Ales K.L., MacKenzie C.R. (1987). A new method of classifying prognostic comorbidity in longitudinal studies: Development and validation. J. Chronic Dis..

[14] von Elm E., Altman D.G., Egger M., Pocock S.J., Gøtzsche P.C., Vandenbroucke J.P., STROBE Initiative (2007). The Strengthening the Reporting of Observational Studies in Epidemiology (STROBE) statement: Guidelines for reporting observational studies. Lancet.

[15] Takeshita T., Tomioka M., Shimazaki Y., Matsuyama M., Koyano K., Matsuda K., Yamashita Y. (2010). Microfloral characterization of the tongue coating and associated risk for pneumonia-related health problems in institutionalized older adults. J. Am. Geriatr. Soc..

[16] van der Maarel-Wierink C.D., Vanobbergen J.N., Bronkhorst E.M., Schols J.M., de Baat C. (2011). Meta-analysis of dysphagia and aspiration pneumonia in frail elders. J. Dent. Res..

[17] Almirall J., Rofes L., Serra-Prat M., Icart R., Palomera E., Arreola V., Clavé P. (2013). Oropharyngeal dysphagia is a risk factor for community-acquired pneumonia in the elderly. Eur. Respir. J..

[18] Manabe T., Teramoto S., Tamiya N., Okochi J., Hizawa N. (2015). Risk factors for aspiration pneumonia in older adults. PLoS ONE.

[19] Ebihara S., Sekiya H., Miyagi M., Ebihara T., Okazaki T. (2016). Dysphagia, dystussia, and aspiration pneumonia in elderly people. J. Thorac Dis..

[20] Ikebe K., Matsuda K., Kagawa R., Enoki K., Okada T., Yoshida M., Maeda Y. (2012). Masticatory performance in older subjects with varying degrees of tooth loss. J. Dent..

[21] Naka O., Anastassiadou V., Pissiotis A. (2014). Association between functional tooth units and chewing ability in older adults: A systematic review. Gerodontology.

[22] Furuya J., Tamada Y., Sato T., Hara A., Nomura T., Kobayashi T., Sakai M., Kondo H. (2016). Wearing complete dentures is associated with changes in the three-dimensional shape of the oropharynx in edentulous older people that affect swallowing. Gerodontology.

[23] Furuta M., Komiya-Nonaka M., Akifusa S., Shimazaki Y., Adachi M., Kinoshita T., Kikutani T., Yamashita Y. (2013). Interrelationship of oral health status, swallowing function, nutritional status, and cognitive ability with activities of daily living in Japanese elderly people receiving home care services due to physical disabilities. Community Dent. Oral Epidemiol..

[24] Iinuma T., Arai Y., Abe Y., Takayama M., Fukumoto M., Fukui Y., Iwase T., Takebayashi T., Hirose N., Gionhaku N. (2015). Denture wearing during sleep doubles the risk of pneumonia in the very elderly. J. Dent. Res..

[25] O’Donnell L.E., Smith K., Williams C., Nile C.J., Lappin D.F., Bradshaw D., Lambert M., Robertson D.P., Bagg J., Hannah V. (2016). Dentures are a Reservoir for Respiratory Pathogens. J. Prosthodont..

[26] Compagnoni M.A., Souza R.F., Marra J., Pero A.C., Barbosa D.B. (2007). Relationship between Candida and nocturnal denture wear: Quantitative study. J. Oral Rehabil..

[27] Baran I., Nalçaci R. (2009). Self-reported denture hygiene habits and oral tissue conditions of complete denture wearers. Arch. Gerontol. Geriatr..

